# Decoupling engineering of formamidinium–cesium perovskites for efficient photovoltaics

**DOI:** 10.1093/nsr/nwac127

**Published:** 2022-07-05

**Authors:** Haoran Chen, Yong Wang, Yingping Fan, Yuetian Chen, Yanfeng Miao, Zhixiao Qin, Xingtao Wang, Xiaomin Liu, Kaicheng Zhu, Feng Gao, Yixin Zhao

**Affiliations:** School of Environmental Science and Engineering, Frontiers Science Center for Transformative Molecules, Shanghai Jiao Tong University, Shanghai 200240, China; Department of Physics, Chemistry and Biology (IFM), Linköping University, Linköping 58183, Sweden; School of Environmental Science and Engineering, Frontiers Science Center for Transformative Molecules, Shanghai Jiao Tong University, Shanghai 200240, China; School of Environmental Science and Engineering, Frontiers Science Center for Transformative Molecules, Shanghai Jiao Tong University, Shanghai 200240, China; School of Environmental Science and Engineering, Frontiers Science Center for Transformative Molecules, Shanghai Jiao Tong University, Shanghai 200240, China; School of Environmental Science and Engineering, Frontiers Science Center for Transformative Molecules, Shanghai Jiao Tong University, Shanghai 200240, China; School of Environmental Science and Engineering, Frontiers Science Center for Transformative Molecules, Shanghai Jiao Tong University, Shanghai 200240, China; School of Environmental Science and Engineering, Frontiers Science Center for Transformative Molecules, Shanghai Jiao Tong University, Shanghai 200240, China; School of Environmental Science and Engineering, Frontiers Science Center for Transformative Molecules, Shanghai Jiao Tong University, Shanghai 200240, China; Department of Physics, Chemistry and Biology (IFM), Linköping University, Linköping 58183, Sweden; School of Environmental Science and Engineering, Frontiers Science Center for Transformative Molecules, Shanghai Jiao Tong University, Shanghai 200240, China; Shanghai Institute of Pollution Control and Ecological Security, Shanghai 200240, China

**Keywords:** formamidinium–cesium, perovskite solar cell, decoupling engineering, sequential cesium incorporation, uniform composition distribution

## Abstract

Although pure formamidinium iodide perovskite (FAPbI_3_) possesses an optimal gap for photovoltaics, their poor phase stability limits the long-term operational stability of the devices. A promising approach to enhance their phase stability is to incorporate cesium into FAPbI_3_. However, state-of-the-art formamidinium–cesium (FA–Cs) iodide perovskites demonstrate much worse efficiency compared with FAPbI_3_, limited by the different crystallization dynamics of formamidinium and cesium, which result in poor composition homogeneity and high trap densities. We develop a novel strategy of crystallization decoupling processes of formamidinium and cesium via a sequential cesium incorporation approach. As such, we obtain highly reproducible, highly efficient and stable solar cells based on FA_1__–_*_x_*Cs*_x_*PbI_3_ (*x* = 0.05–0.16) films with uniform composition distribution in the nanoscale and low defect densities. We also revealed a new stabilization mechanism for Cs doping to stabilize FAPbI_3_, i.e. the incorporation of Cs into FAPbI_3_ significantly reduces the electron–phonon coupling strength to suppress ionic migration, thereby improving the stability of FA–Cs-based devices.

## Teaser

Sequential Cs incorporation strategy is developed to decouple crystallization of FACs perovskite with reduced electron-phonon coupling, resulting in highly stable FACs tri-iodide perovskite photovoltaics with record efficiency.

## INTRODUCTION

Metal–halide perovskites with superior photophysical properties and low-cost solution technology have emerged as promising candidates for different optoelectronic devices, including solar cells, light-emitting diodes, etc. [1–6]*.* For perovskite solar cells (PSCs), the certified power-conversion efficiency (PCE) has reached 25.7%, which is comparable to the current commercial crystalline silicon solar cells. ABX_3_ perovskites with tailoring compositions, where A is an organic or inorganic cation, B is a metal cation and X is a halide anion, have been attempted for high efficiency and stable photovoltaic devices. Among these, formamidinium lead iodide (FAPbI_3_) has exhibited great potential as the absorber layer, due to its optimal band gap of ∼1.5 eV and excellent thermal stability [7–9].

However, the photoactive FAPbI_3_ black phase would easily transform into a non-photoactive yellow δ-FAPbI_3_ phase at room temperature, especially under humid conditions [10,11]. The poor phase stability challenges both the efficiency and long-term stability of the PSCs based on FAPbI_3_ [12,13]. It is generally believed that the phase instability of FAPbI_3_ perovskites originates from its unsuitable tolerant factor. To address this problem, alloying FA^+^ with MA^+^/Cs^+^ cations or partially substituting I^−^ with Br^−^ ions has been employed to tune the tolerant factor [14,15]. The resulting mixed-ion FA-based perovskites exhibit improved resistance to phase transition.

Among these different alloying approaches, formamidinium–cesium mixed-cation pure iodide (FA_1__–_*_x_*Cs*_x_*PbI_3_) perovskites are particularly promising, because they avoid the concerns about volatile MA cations and phase segregation induced by mixed halide ions (Br–I) [16–20]. However, because of the complex crystallization kinetics of formamidinium and cesium, these pure iodide FA–Cs perovskites fabricated by one-step crystallization suffer from poor composition homogeneity and high defects/traps densities [21,22]. The PSCs based on these films are therefore facing relatively low efficiencies. Especially, strong non-radiative recombination in all reported FA–Cs-based PSCs limited the open-circuit voltage (*V*_oc_) of the resulting devices [23–25].

Herein, we develop a novel sequential Cs incorporation (SCI) strategy to decouple the crystallization processes of formamidinium and cesium, and achieve highly efficient pure iodide FA_1__–_*_x_*Cs*_x_*PbI_3_ (*x *= 0.05–0.16) perovskites (denoted as SCI-FA_1__–_*_x_*Cs*_x_*PbI_3_). The ratio of FA and Cs in FA_1__–_*_x_*Cs*_x_*PbI_3_ can be straightforwardly tuned by introducing different concentrations of cesium formate (HCOOCs) solution on the FA-based perovskite precursor film during the SCI process. A unique feature of our SCI-FA_1__–_*_x_*Cs*_x_*PbI_3_ perovskites is the uniform distributions of Cs cations, in contrast to their poor uniformity in typical one-step (1S) crystallized FA–Cs perovskite films (denoted as 1S-FA_1__–_*_x_*Cs*_x_*PbI_3_). As a result, the champion SCI-FA_0.91_Cs_0.09_PbI_3_ PSCs yield a record PCE of 24.7% (certified 23.8%) with improved *V*_oc_ and fill factor, which is the highest value for the pure iodide FA_1__–_*_x_*Cs*_x_*PbI_3_ perovskites. Compared with FAPbI_3_, the SCI-FA_0.91_Cs_0.09_PbI_3_ perovskite shows reduced electron–phonon coupling and lattice fluctuations, which suppress the formation of iodide-rich clusters and finally contribute to the excellent operational stability of the FA_0.91_Cs_0.09_PbI_3_-based PSCs.

## RESULT AND DISCUSSION

**Figure 1.**
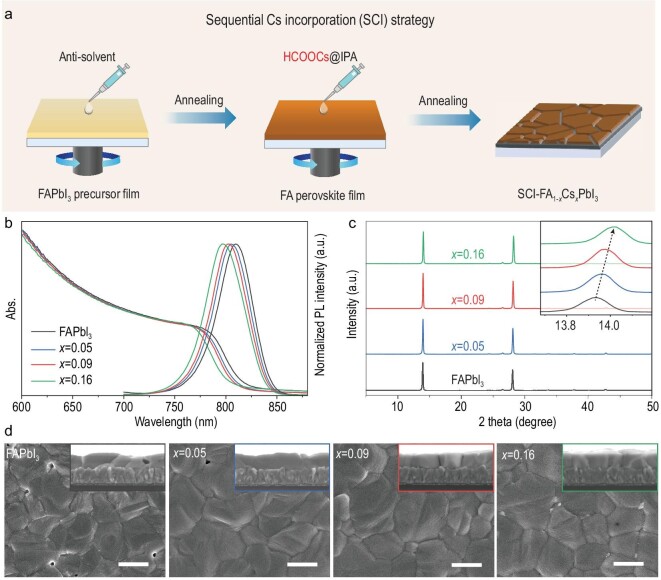
Spectroscopic, structural and morphological characterizations of SCI-FA_1__–_*_x_*Cs*_x_*PbI_3_ films. (a) Schematic diagram of the SCI-FA_1__–_*_x_*Cs*_x_*PbI_3_ perovskite films fabricated by decoupling the crystallization processes of formamidinium and cesium. (b) UV–vis absorption and normalized PL spectra and (c) XRD patterns of FAPbI_3_ and SCI-FA_1__–_*_x_*Cs*_x_*PbI_3_ perovskites. Inset pattern corresponding to the characteristic peaks of (001) perovskite crystal planes. (d) Top-surface SEM images of FAPbI_3_ and SCI-FA_1__–_*_x_*Cs*_x_*PbI_3_ perovskite films. The inset presents the cross-sectional morphology of the corresponding perovskite films. Scale bars: 1 μm.

Figure 1a shows the schematic diagram of SCI-FA_1__–_*_x_*Cs*_x_*PbI_3_ perovskite films prepared by decoupling the crystallization processes of formamidinium and cesium. A FAPbI_3_ precursor film was first deposited by a typical anti-solvent method followed by annealing for 1 min. The Cs cation is sequentially introduced onto the FA perovskite film by spin-coating HCOOCs isopropanol (IPA) solution, followed by further annealing. For comparison, we employed different concentrations of HCOOCs solution (2.5, 5 and 10 mg mL^–1^) to fabricate SCI-FA_1__–_*_x_*Cs*_x_*PbI_3_ perovskites. The final ratios of incorporated Cs in the above SCI-FA_1__–_*_x_*Cs*_x_*PbI_3_ perovskite films, i.e. the value of *x*, are 0.05, 0.09 and 0.16, as confirmed by inductively coupled plasma–mass spectrometry (ICP–MS) analysis (Supplementary Table 1). The corresponding SCI-FA_1__–_*_x_*Cs*_x_*PbI_3_ perovskite films are noted as *x *= 0.05, *x *= 0.09 and *x *= 0.16 in Fig. 1.

Optical and structural measurements of perovskite films indicate that Cs^+^ from HCOOCs has successfully been incorporated into the lattice of FAPbI_3_ perovskites. Figure 1b shows the ultraviolet–visible (UV–vis) spectra of SCI-FA_1__–_*_x_*Cs*_x_*PbI_3_ perovskite films, in which the absorption edges of SCI-FA_1__–_*_x_*Cs*_x_*PbI_3_ perovskites strongly depend on the amount of Cs^+^ incorporation. When *x* increases from 0 to 0.16, the absorption edges of SCI-FA_1__–_*_x_*Cs*_x_*PbI_3_ perovskites gradually blue-shift from 816 to 802 nm, and the corresponding photoluminescence (PL) peaks shift from 809 to 797 nm. The X-ray diffraction (XRD) measurements are carried out to investigate the crystal structure evolution of SCI-FA_1__–_*_x_*Cs*_x_*PbI_3_ perovskites (Fig. 1c). All the SCI-FA_1__–_*_x_*Cs*_x_*PbI_3_ perovskites exhibit stronger peak intensity than the pure FAPbI_3_ at around both 14° and 28°, corresponding to (001) and (002) perovskite crystal planes. The inset image of Fig. 1c shows that the peak between 13.8° and 14.1° shifts to a higher degree, indicating that Cs ions are incorporated into the perovskite lattice. The lattice parameter decreases with increasing the amount of Cs (Supplementary Fig. 1), further confirming the successful mixing of Cs^+^ in the perovskite lattice. The tolerance factor of SCI-FA_1__–_*_x_*Cs*_x_*PbI_3_ perovskites is also reduced compared with pure FA perovskite, potentially contributing to an stable perovskite structure (Supplementary Fig. 2).

The Cs incorporation also significantly improves the film morphologies (Fig. 1d). All SCI-FA_1__–_*_x_*Cs*_x_*PbI_3_ films show enlarged and pinhole-free grains compared with the FAPbI_3_ film, which shows coarse grains and pinholes. As shown in the cross-sectional scanning electron microscopy (SEM) images, SCI-FA_1__–_*_x_*Cs*_x_*PbI_3_ perovskite films (550–600 nm) with vertical growth of grains benefit efficient charge extraction.

By adopting these Cs-incorporated perovskites as light absorber layers, we fabricate PSCs with a configuration of fluorine-doped tin oxide (FTO)/electron-transport layer/perovskite/hole-transport layer/Au. All SCI-FA_1__–_*_x_*Cs*_x_*PbI_3_ (*x *= 0.05, 0.09, 0.16)-based PSCs exhibit improved device efficiency compared with the FAPbI_3_-based devices (Supplementary Fig. 3). Considering that *x *= 0.09 provides the optimal photovoltaic (PV) performance, we then chose this composition (denoted as SCI-FA_0.91_Cs_0.09_PbI_3_) for detailed investigations on Cs incorporation and its role on film and device properties.

X-ray photoelectron spectroscopy spectra are conducted to explore the effect of SCI on the elements and their chemical states in perovskite films. All core-level peaks are assigned to Cs, Pb, N and I (Supplementary Fig. 4) elements. The characteristic Cs signals in the SCI-FA_0.91_Cs_0.09_PbI_3_ perovskite locate at 738.5 and 724.7 eV, and show a 1.1-eV shift compared with the Cs in HCOOCs. Such a large shift is attributed to the formation of chemical bonds between Cs^+^ and [PbI_6_]^4−^. For the Pb 4*f* spectra in FAPbI_3_, two peaks corresponding to Pb 4*f*_7/2_ and Pb 4*f*_5/2_ are observed at 138.3 and 143.2 eV. However, in the SCI-FA_0.91_Cs_0.09_PbI_3_ perovskite, both Pb 4*f*_7/2_ and Pb 4*f*_5/2_ shift by 0.1 eV toward higher binding energy, originating from the stronger bond energies between Cs^+^ and [PbI_6_]^4−^ than that between FA^+^ and [PbI_6_]^4−^. Additionally, the N and I elements representing the formamidine component have undergone a small shift. These results further confirm that Cs has been successfully incorporated into the FAPbI_3_ perovskite lattice to form SCI-FA_0.91_Cs_0.09_PbI_3_ perovskite.

For comparison, we also fabricate one-step crystallized 1S-FA_1__–_*_x_*Cs*_x_*PbI_3_, where Cs^+^ (from HCOOCs) is directly mixed with FA^+^ in the perovskite precursor. For 1S-FA_1__–_*_x_*Cs*_x_*PbI_3_ PSCs (Supplementary Fig. 5), the optimal efficiency is also obtained from the *x *= 0.09 sample, which hence will be used as the control sample for comparison with SCI-FA_0.91_Cs_0.09_PbI_3_. The absorption edge, characteristic XRD peaks and surface morphologies of the 1S-FA_0.91_Cs_0.09_PbI_3_ perovskite films are consistent with SCI-FA_0.91_Cs_0.09_PbI_3_ perovskite (Supplementary Fig. 6).

**Figure 2.**
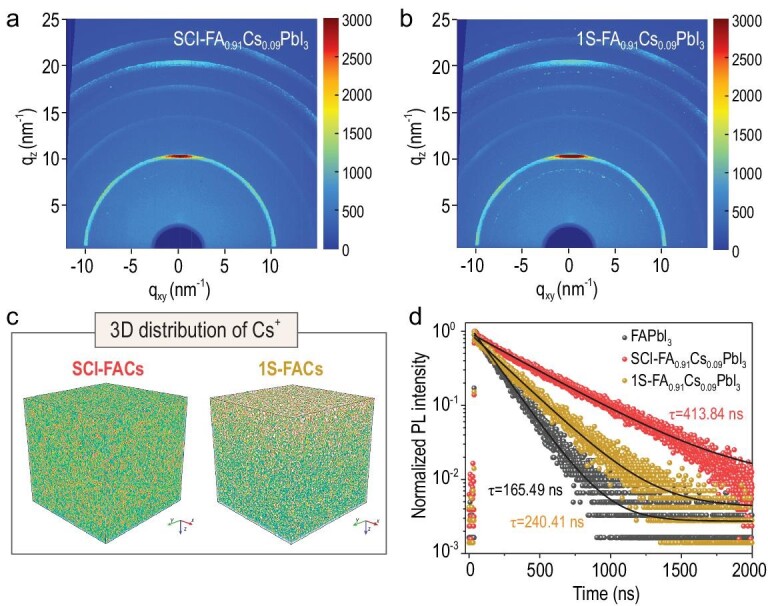
Characterizations of FA_0.91_Cs_0.09_PbI_3_ perovskite films. GIWAXS data of (a) SCI-FA_0.91_Cs_0.09_PbI_3_ and (b) 1S-FA_0.91_Cs_0.09_PbI_3_ films. (c) 3D distribution in the SCI-FA_0.91_Cs_0.09_PbI_3_ and 1S-FA_0.91_Cs_0.09_PbI_3_ film by ToF-SIMS analysis, where the signal intensity in two kinds of perovskites is normalized. (d) TRPL decay curves of FAPbI_3_, SCI-FA_0.91_Cs_0.09_PbI_3_ and 1S-FA_0.91_Cs_0.09_PbI_3_ perovskite films.

The synchrotron-based grazing-incidence wide-angle X-ray scattering (GIWAXS) and time-of-flight secondary ion mass spectrometry (ToF-SIMS) measurements are further employed to explore the crystal structure and internal composition in perovskite films. As shown in Fig. 2a and b, there is a signal ring at *q*_xy_ = 8.8 nm^−1^ corresponding to δ-FAPbI_3_ in 1S-FA_0.91_Cs_0.09_PbI_3_ perovskite, while the GIWAXS result of SCI-FA_0.91_Cs_0.09_PbI_3_ exhibits high phase purity and obvious crystal orientation without any obvious phase impurities. Since the distribution of Cs in SCI-FA_1__–_*_x_*Cs*_x_*PbI_3_ perovskite has a significant effect on both the phase stability and traps/defects [22,26], we proceed to investigate the distribution of Cs in the SCI-FA_0.91_Cs_0.09_PbI_3_ and 1S-FA_0.91_Cs_0.09_PbI_3_ perovskites. Both energy-dispersive spectroscopy mapping and ToF-SIMS have established that the incorporated Cs^+^ cations homogenously distribute in the surface and bulk of SCI-FA_0.91_Cs_0.09_PbI_3_ perovskite, which is completely different from the inhomogeneous Cs^+^ cation distributions in 1S-FA_0.91_Cs_0.09_PbI_3_ perovskite (Fig. 2c and Supplementary Fig. 7). The Cs aggregation at the top surface of 1S-FA_0.91_Cs_0.09_PbI_3_ reveals the phase separation in 1S-FA_0.91_Cs_0.09_PbI_3_, which is consistent with the GIWAXS data. Such uniform distribution of Cs ions in the SCI-FA_0.91_Cs_0.09_PbI_3_ perovskite originates from the decoupled crystallization kinetics by the SCI strategy, which is beneficial for enhancing the phase stability and reducing defects/traps density. Different from Cs^+^ cations, other ions, including FA^+^, Pb^2+^ and I^−^, are uniformly distributed throughout both the SCI-FA_0.91_Cs_0.01_PbI_3_ and 1S-FA_0.91_Cs_0.09_PbI_3_ perovskite films (Supplementary Fig. 8). Although most of the HCOO^−^ anions escape from the FA_0.91_Cs_0.09_PbI_3_ perovskite during the annealing process, residual traces of HCOO^−^ still exist at the bottom interface between the perovskite and the substrate (Supplementary Fig. 9), which can further passivate the defects on the bottom interface and improve the charge carriers’ dynamics in the devices [27,28].

Uniform incorporation of Cs ions into FAPbI_3_ has two positive effects: enhanced phase stability and decreased trap/defect densities. The enhanced phase stability is evidenced from the absence of color/structural changes under humid conditions. Under 60% relative humidity, the SCI-FA_0.91_Cs_0.09_PbI_3_ perovskite maintains the black phase for 7 days without any changes (Supplementary Fig. 10), showing significant improvement compared with FAPbI_3_. The decreased trap/defect densities are demonstrated from photophysical measurements. The photoluminescence (PL) intensities of SCI-FA_0.91_Cs_0.09_PbI_3_ perovskites are much stronger than those of 1S-FA_0.91_Cs_0.09_PbI_3_ and pure FA perovskite films (Supplementary Fig. 11). In addition, the time-resolved PL (TRPL) spectra in Fig. 2d show that the PL lifetime (*τ*) of SCI-FA_0.91_Cs_0.09_PbI_3_ is much longer (413.84 ns) than that of 1S-FA_0.91_Cs_0.09_PbI_3_ (240.41 ns) and FAPbI_3_ (165.49 ns). Enhanced PL intensity and improved PL lifetime indicate that non-radiative recombination is suppressed in SCI-FA_0.91_Cs_0.09_PbI_3_, attributed to decreased trap densities. Consistently, we calculated the Urbach energy (*E*_u_) according to the equation: *A* = A_0_ exp(*E*/*E*_u_), where *A* is the absorbance, A_0_ is a constant for data fitting and *E* is the excitation energy. The Urbach energy (Supplementary Fig. 12) is decreased from 22.2 meV in FAPbI_3_ and 21.6 meV in 1S-FA_0.91_Cs_0.09_PbI_3_ to 18.3 meV in SCI-FA_0.91_Cs_0.09_PbI_3_. The smaller Urbach energy in SCI-FA_0.91_Cs_0.09_PbI_3_ corresponds to a lower density of trap states. These results suggest that, with sequential incorporation of Cs^+^, the SCI-FA_0.91_Cs_0.09_PbI_3_ perovskite exhibits significantly reduced non-radiative recombination via defects/traps.

**Figure 3.**
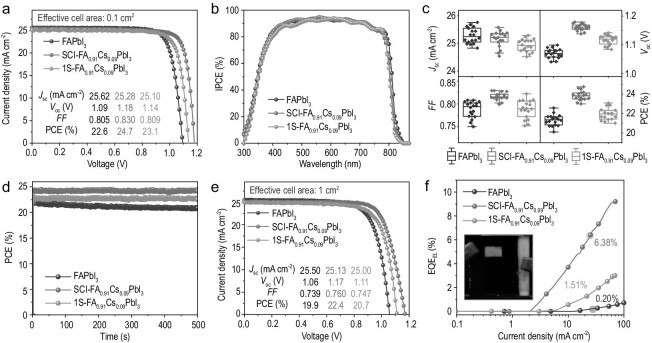
Photovoltaic and device characterization. (a) The *J–V* curves of the champion devices of FAPbI_3_, SCI-FA_0.91_Cs_0.09_PbI_3_ and 1S-FA_0.91_Cs_0.09_PbI_3_ PSCs with 0.1 cm^2^ of effective cell area. (b) IPCE spectra of FAPbI_3_, SCI-FA_0.91_Cs_0.09_PbI_3_- and 1S-FA_0.91_Cs_0.09_PbI_3_-based PSCs. (c) The PV performance distribution of FAPbI_3_-, SCI-FA_0.91_Cs_0.09_PbI_3_- and 1S-FA_0.91_Cs_0.09_PbI_3_-based PSCs from 18 devices, respectively. (d) Steady-state efficiency of FAPbI_3_ and SCI-FA_0.91_Cs_0.09_PbI_3_ and 1S-FA_0.91_Cs_0.09_PbI_3_ PSCs. (e) *J–V* characteristics of PSCs based on FAPbI_3_, SCI-FA_0.91_Cs_0.09_PbI_3_ and 1S-FA_0.91_Cs_0.09_PbI_3_ with 1 cm^2^ of effective cell area under simulated AM 1.5-G solar illumination of 100 mW cm^–2^ in the reverse scan. (f) EQE_EL_ of FAPbI_3_-, SCI-FA_0.91_Cs_0.09_PbI_3_- and 1S-FA_0.91_Cs_0.09_PbI_3_-based PSCs vs. the current density.

Benefitting from these advantages of crystallization decoupling engineering, the resulting SCI-FA_0.91_Cs_0.09_PbI_3_ perovskite shows much enhanced device performance. Figure 3a compares the current density–voltage (*J*–*V*) characteristics of champion PSCs based on FAPbI_3_, SCI-FA_0.91_Cs_0.09_PbI_3_ and 1S-FA_0.91_Cs_0.09_PbI_3_ perovskites, respectively. The SCI-FA_0.91_Cs_0.09_PbI_3_-based PSC exhibits an impressive PCE of 24.7% compared with 22.6% for FAPbI_3_, representing a new record for pure iodide FA_1__–_*_x_*Cs*_x_*PbI_3_-based PSCs (Supplementary Table 2). A certified PCE of 23.8% with negligible hysteresis has been obtained in the SCI-FA_0.91_Cs_0.09_PbI_3_-based PSCs (Supplementary Fig. 13). The most striking difference is the *V*_oc_, which increases from 1.09 V in FAPbI_3_ to 1.18 V in SCI-FA_0.91_Cs_0.09_PbI_3_. The counterpart PSCs based on 1S-FA_0.91_Cs_0.09_PbI_3_ perovskite yield a lower efficiency of 23.1%. This comparison indicates that our crystallization decoupling engineering is beneficial for enhancing SCI-FA_1__–_*_x_*Cs*_x_*PbI_3_-based PSCs performance. The incident photon to electron conversion efficiency (IPCE) (Fig. 3b) is similar for both devices, with a high value of >90% in the wavelength range of 450–650 nm. The short-circuit current density (*J*_sc_) of the SCI-FA_0.91_Cs_0.09_PbI_3_ device is slightly decreased compared with the FAPbI_3_ device, mainly due to slight increase in the band gap upon Cs incorporation. Figure 3c compares the PV parameters of FAPbI_3_-, SCI-FA_0.91_Cs_0.09_PbI_3_- and 1S-FA_0.91_Cs_0.09_PbI_3_-based PSCs for 18 devices, respectively, indicating that sequential Cs incorporation also improves the device reproducibility. In addition, the SCI-FA_0.91_Cs_0.09_PbI_3_-based PSCs exhibit a smaller hysteresis (Supplementary Figs 13 and 14), resulting in a stabilized output power of 24.4% (Fig. 3d).

In addition to small-area PSCs, the large-area PSCs based on these sequential Cs-incorporated perovskite films also exhibit significantly improved device performance. The champion SCI-FA_0.91_Cs_0.09_PbI_3_ device, fabricated on 2.5 × 2.5 cm^2^ substrates with an effective cell area of 1 cm^2^ (Fig. 3e), displays a PCE of 22.4%, which is far higher than the FAPbI_3_ (∼19.9%) and 1S-FA_0.91_Cs_0.09_PbI_3_ (∼20.7%)-based devices.

The significantly enhanced *V*_oc_ of the SCI-FA_0.91_Cs_0.09_PbI_3_ device is mainly due to suppressed non-radiative recombination, which can be quantified by measuring the external quantum efficiency of electroluminescence (EQE_EL_) values [29]. As shown in Fig. 3f, at the injection current densities corresponding to *J*_sc_, the EQE_EL_ value of the SCI-FA_0.91_Cs_0.09_PbI_3_ device is 6.38%, while that of the FAPbI_3_ device is 0.16%. We calculate the voltage losses due to non-radiative recombination (Δ*V*_ocnon-rad_,) based on the formula [30]:

$$\begin{equation*}
\Delta \ \!\!{V}_{{\rm{oc}},{\rm{non}} - {\rm{rad}}} = \ - \frac{{{\rm{k}}T}}{{\rm{q}}}\ln {\rm{EQ}}{{\rm{E}}}_{{\rm{EL}}},
\end{equation*}$$

where k, *T* and *q* represent the Boltzmann constant, temperature and elementary electric charge, respectively. The difference in Δ*V*_ocnon-rad_, (0.09 V) matches well with the difference of device *V*_oc_ (0.09 V).

Suppressed non-radiative recombination in the SCI-FA_0.91_Cs_0.09_PbI_3_ device is consistent with previous photophysical measurements on the films, which indicate that the sequential Cs incorporation can reduce the defects/traps. Further measurements on the devices also reach similar conclusions. The trap-filled limiting voltage in the space-charge limited current measurements decreases from 0.13 V in the FAPbI_3_ device to 0.09 V in SCI-FA_0.91_Cs_0.09_PbI_3_ device (Supplementary Fig. 15), indicating suppressed traps/defects upon Cs sequential incorporation [31,32]. These results are also consistent with transient photovoltage (TPV) decay and transient photocurrent (TPC) decay results (Supplementary Fig. 16), which show slower TPV decay (indicating longer recombination lifetime) and quicker TPC decay (indicating fewer trapping effects) in the SCI-FA_0.91_Cs_0.09_PbI_3_ device [33,34].

**Figure 4.**
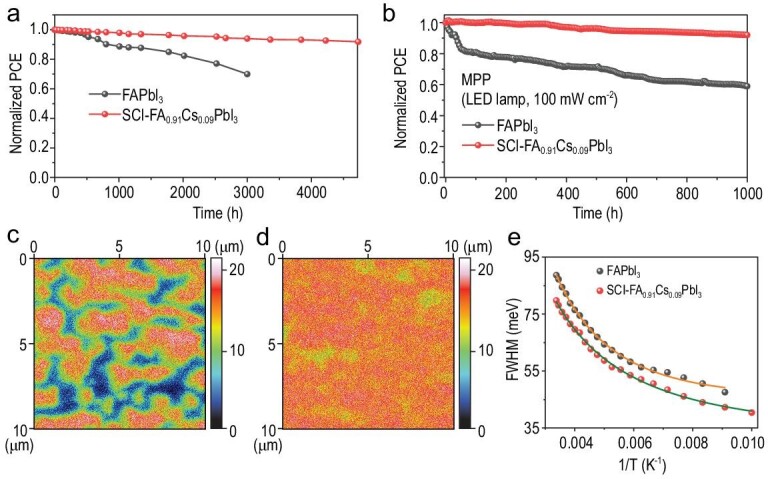
Stability of the SCI-FA_0.91_Cs_0.09_PbI_3_ PSCs. (a) The shelf-life stability of unencapsulated FAPbI_3_ and SCI-FA_0.91_Cs_0.09_PbI_3_ PSCs. (b) The long-term operational stability of unencapsulated FAPbI_3_ and SCI-FA_0.91_Cs_0.09_PbI_3_ PSCs. The 2D I^−^ distribution in the middle of the perovskite layer (at ∼300 nm) for PSCs based on (c) FAPbI_3_ and (d) SCI-FA_0.91_Cs_0.09_PbI_3_ perovskites after 240 h of operational stability test at their MPP by ToF-SIMS. (e) Fit of the FWHM of the PL spectra vs. temperature.

In addition to improved PV performance, the SCI-FA_0.91_Cs_0.09_PbI_3_ device also shows significantly enhanced stability. We first measure the shelf life by storing the unencapsulated devices in dark at 25°C and 20% relative humidity. Figure 4a shows that the PCE of the FAPbI_3_ device decreases by ∼30% after 3000 h of aging, whereas the SCI-FA_0.91_Cs_0.09_PbI_3_ device shows a degradation of only 10% over 4500 h of aging. We then investigate the long-term operational stability of the PSCs by aging the unencapsulated devices under a nitrogen atmosphere, using maximum power point (MPP) tracking under simulated 1-sun conditions. As shown in Fig. 4b, the SCI-FA_0.91_Cs_0.09_PbI_3_ based PSCs retains >90% of the initial PCE while the FAPbI_3_ device maintains only 60% PCE after 1000 h of continuous illumination (Supplementary Fig. 17). Especially, the sharp decline of the efficiency in FAPbI_3_ PSCs in the initial stage should be attributed to the intrinsic instability of the FAPbI_3_ perovskite layer and potential severe ion migration in the FAPbI_3_ device.

A main reason for enhanced-stability PSCs is attributed to suppressed ionic migration. In Fig. 4c and d, we compare the I^−^ ions distribution at the ∼300-nm depth of the perovskite layer for PSCs based on FAPbI_3_ and SCI-FA_0.91_Cs_0.09_PbI_3_ after 240-h MPP test. In the FAPbI_3_-based device, strong aggregation of I^−^ clusters is observed in the perovskite absorber layers; in contrast, I^−^ ions distribute uniformly in the SCI-FA_0.91_Cs_0.09_PbI_3_-based devices. This sharp contract indicates that the ionic migration in the SCI-FA_0.91_Cs_0.09_PbI_3_ is much suppressed upon Cs sequential incorporation.

Suppressed ionic migration in SCI-FA_0.91_Cs_0.09_PbI_3_ is consistent with suppressed electron–phonon coupling upon Cs incorporation. Figure 4e shows the full-width half-maximum (FWHM) of the PL peak of SCI-FA_0.91_Cs_0.09_PbI_3_ and FAPbI_3_ perovskites (Supplementary Fig. 16) ranging from 110 to 296 K. The wide broadening of the PL linewidth in FAPbI_3_ perovskites arises from strong electron–phonon coupling [35,36]. The electron–phonon interaction is dominated by high energy longitudinal optical (LO) phonons in the high-temperature region, where the measured FWHM data could be fitted by the Boson model (Fig. 4c, Supplementary Fig. 18 and Supplementary Table 3). Compared with FAPbI_3_, both the electron–LO phonon coupling coefficient (*Γ*_LO_) and LO phonon energy (*hω*) in the SCI-FA_0.91_Cs_0.09_PbI_3_ are significantly reduced, indicating that the fluctuation of the PbI_6_ octahedra cage in SCI-FA_0.91_Cs_0.09_PbI_3_ is associated with much smaller energies upon the Cs sequential incorporation. This is consistent with the previous theoretical investigations, which indicate that mixed A-site cations could reduce the lattice fluctuations in halide perovskites [37]. As such, the suppressed lattice fluctuations and electron–phonon coupling in SCI-FA_0.91_Cs_0.09_PbI_3_ rationalize suppressed ionic migration and hence enhanced stability in SCI-FA_0.91_Cs_0.09_PbI_3_ PSCs, which agree with previous research results that the suppressed lattice fluctuations and reduced electron–phonon coupling could suppressed the formation of iodide-rich clusters to improve the stability of halide perovskites [38,39].

## CONCLUSION

In summary, we successfully develop a novel SCI strategy to tackle the critical challenge of different crystallization dynamics of different cations in developing FA_1__–_*_x_*Cs*_x_*PbI_3_ perovskite PSCs. The resulting pure iodide SCI-FA_1__–_*_x_*Cs*_x_*PbI_3_ perovskites show more uniform composition distribution and reduced defects/traps density than FAPbI_3_ and one-step crystallized 1S-FA_0.91_Cs_0.09_PbI_3_. Compared with FAPbI_3_, the SCI-FA_0.91_Cs_0.09_PbI_3_ exhibits reduced electron–phonon coupling and lattice fluctuations, minimizing ion migration and hence enhancing the stability. As such, we have been able to achieve highly stable PSCs with a high efficiency of 24.7%, which is a record for SCI-FA_1__–_*_x_*Cs*_x_*PbI_3_ PSCs. This work opens up new possibilities to develop high-quality mixed-cation perovskites, presenting a milestone towards the development of highly efficient and highly stable perovskites for various applications, including solar cells, light-emitting diodes and lasers.

## METHODS

### Materials

Lead iodide (PbI_2_, 99.9985%), cesium formate (HCOOCs, 98%), methylamine hydrochloride (MACl, 99%) and tin (IV) oxide colloid precursor (SnO_2_, 15% in H_2_O colloidal dispersion) were purchased from Alfa Aesar. Formamidinium iodide (FAI) was purchased from Xi’an Polymer Light Technology Corp. Other materials were purchased from Sigma-Aldrich and used as received without any purification. *N*,*N*-dimethylformamide (DMF, anhydrous, 99.8%), dimethyl sulfoxide (DMSO, anhydrous, 99.7%), chlorobenzene (anhydrous, 99.8%) and isopropanol alcohol (IPA, 99.5%) were purchased from J&K Scientific Ltd. Ammonium solution (AR, 25%–28%) was purchased from Aladdin.

### Device fabrication

A compact TiO_2_ layer (20 nm) was deposited using the spray pyrolysis method using a titanium bis(ethyl acetoacetate)-diisopropoxide/1-butanol solution (1 : 9 volume ratio). The cleaned patterned fluorine-doped tin oxide (FTO, 7 Ω sq^–1^) substrate was placed on a 450°C plate during the spray process followed by 1 h of annealing. The SnO_2_ colloid precursor/ammonium solution (1 : 9 volume ratio) was spin-coated on a TiO_2_ layer at 3000 rpm for 30 s followed by annealing for 30 min at 180°C. The SCI-FA_1__–_*_x_*Cs*_x_*PbI_3_ perovskite precursor was prepared by mixing PbI_2_, FAI and MACl (30 mol%) in DMF : DMSO = 9:1 solution to form 1.5 M FAPbI_3_ precursor. The perovskite precursor films were deposited by spin-coating on FTO/TiO_2_/SnO_2_ substrate at 5000 rpm for 15 s. During spin-coating, 150–200 μL of chlorobenzene was dripped at the end of 10 s. For the SCI perovskite samples, the perovskite film was first annealed for 1 min at 150°C to obtain a light-brown FA perovskite film. Different concentrations of HCOOCs/IPA solution were dropped on the FAPbI_3_ precursor films then spin-coated at 3000 rpm for 30 s and further annealed at 150°C for 9 min and 100°C for 10 min. The 1S-FA_1__–_*_x_*Cs*_x_*PbI_3_ perovskite was prepared by mixing PbI_2_, FAI, MACl (30 mol%) and x mol% HCOOCs in DMF : DMSO = 9 : 1 solution to form 1.5 M FA–Cs precursor. The perovskite precursor films were deposited by spin-coating on FTO/TiO_2_/SnO_2_ substrate at 5000 rpm for 15 s. During spin-coating, 150–200 μL of chlorobenzene was dripped at the end of 10 s. The perovskite films annealed at 150°C for 10 min and 100°C for 10 min. The spiro-OMeTAD layer was spin-coated by dissolving 90 mg of spiro-OMeTAD in 1 mL of chlorobenzene and mixing with 39.5 μL of 4-tert-butylpyridine (TBP), 23 μL of Li-bis(trifluoromethanesulfonyl) imide (Li-TFSI)/acetonitrile (520 mg/mL) and 10 μL of tris(2-(1h-pyrazol-1-yl)-4-tert-butylpyridine)-cobalt(III)tris(bis(trifluoromethylsulfonyl)imide) (FK209)/acetonitrile (375 mg mL^–1^) at 4000 rpm for 25 s. Finally, a 80-nm thick gold electrode was thermal evaporated on the spiro-OMeTAD layer. The perovskite films fabrication was operated in a dry box with <15% humidity.

## Supplementary Material

nwac127_Supplemental_file
