# Supplementary material for: Decoupling engineering of formamidinium–cesium perovskites for efficient photovoltaics
Source: Natl Sci Rev. 2022 Jul 5;9(10):nwac127. doi: 10.1093/nsr/nwac127 (PMC9522398; doi:10.1093/nsr/nwac127)
Supplement: nwac127_Supplemental_file [file nwac127_supplemental_file.docx]

Supplementary Information for

**Decoupling Engineering of Formamidinium-Cesium Perovskites for Efficient Photovoltaics**

Haoran Chen^1,†^, Yong Wang^2,†^, Yingping Fan^1,†^, Yuetian Chen^1^, Yanfeng Miao^1^, Zhixiao Qin^1^, Xingtao Wang^1^, Xiaomin Liu^1^, Kaicheng Zhu^1^, Feng Gao^2,^*, Yixin Zhao^1,3,^*

1.School of Environmental Science and Engineering, Frontiers Science Center for Transformative Molecules, Shanghai Jiao Tong University, Shanghai 200240, China;

2. Department of Physics, Chemistry and Biology (IFM), Linköping University, 581 83 Linköping, Sweden;

3.Shanghai Institute of Pollution Control and Ecological Security, Shanghai 200240, China

***Corresponding authors.** E-mails: yixin.zhao@sjtu.edu.cn; [feng.gao@liu.se](mailto:feng.gao@liu.se)

^†^Equally contributed to this work.

This file contains

Supplementary Figures 1-18

Supplementary Tables 1-3

**Film Characterization**

The XRD patterns were measured by Shimadzu XRD-6100 diffractometer with a Cu Kα source. The UV-vis spectra of the films were measured using a Cary-60 UV-vis spectrophotometer. Steady-state and time resolved photoluminescence and were measured by FLS 1000 photoluminescence spectrometer with an excitation wavelength of 450 nm. The SEM and EDS images were carried out using JSM-7800F Prime scanning electron microscope. The XPS spectra were carried out on AXIS Ultra DLD spectrometer by using an Al-Ka X‐ray source. GIWAXS measurements were performed at the BL14B1 beamline of the Shanghai Synchrotron Radiation Facility (SSRF) with a beam wavelength of 0.12398 nm. The ToF-SIMS analysis were measured by TOF SIMS 5-100 (ION-TOF GmbH, Germany), the primary ion beam is Bi nanoprobe, and sputter beam is GCIB (gas cluster ion beam). Temperature-dependent PL spectra of the FAPbI_3_ and SCI-FA_0.91_Cs_0.09_PbI_3_ thin films at 10K interval temperature were recorded by the Andor spectrometer under the same excitation light (450 nm, 120 μW cm^-2^). The elemental content in the perovskite thin films was evaluated by inductively coupled plasma mass spectrometry (ICP-MS, iCAP Q). The temperature was initially decreased to 100 K with helium compressor, and gradually heat the sample space to 296 K.

**Device characterization**

J-V characteristics of the devices were measured with a Keithley 2401 source meter under the simulated AM 1.5G illumination (100 mW cm^-2^) using an Enlitech 3A light source (reserve scan: 1.2 V ─ (−0.1 V), forward scan: 0.1 V ─ 1.2 V, scan rate: 0.05 V S^−1^). The aperture area of non-reflective metal masks in J-V measurement are 0.1 cm^2^ and 1 cm^2^. The IPCE spectra was performed by Enlitech QE-3011 system. The EQE_EL_ was measured by a Keithley 2400 source meter. The emitted light was collected by an integration sphere coupled with a fiber spectrometer (QE 65 Pro, Ocean Optics). The TPV and TPC measurement were obtained by Zahner electrochemical workstation and were measured by a microsecond pulse of a white light incident on solar cells under short-circuit condition and open-circuit condition. The shelf-life stability of the devices was carried out in a nitrogen glove box without encapsulation. The operational stability test of the devices was measured by biasing the devices under continuous illumination of LED (100 mW cm^−2^) at MPP in N_2_ filled glove box (~65°C) without encapsulation.


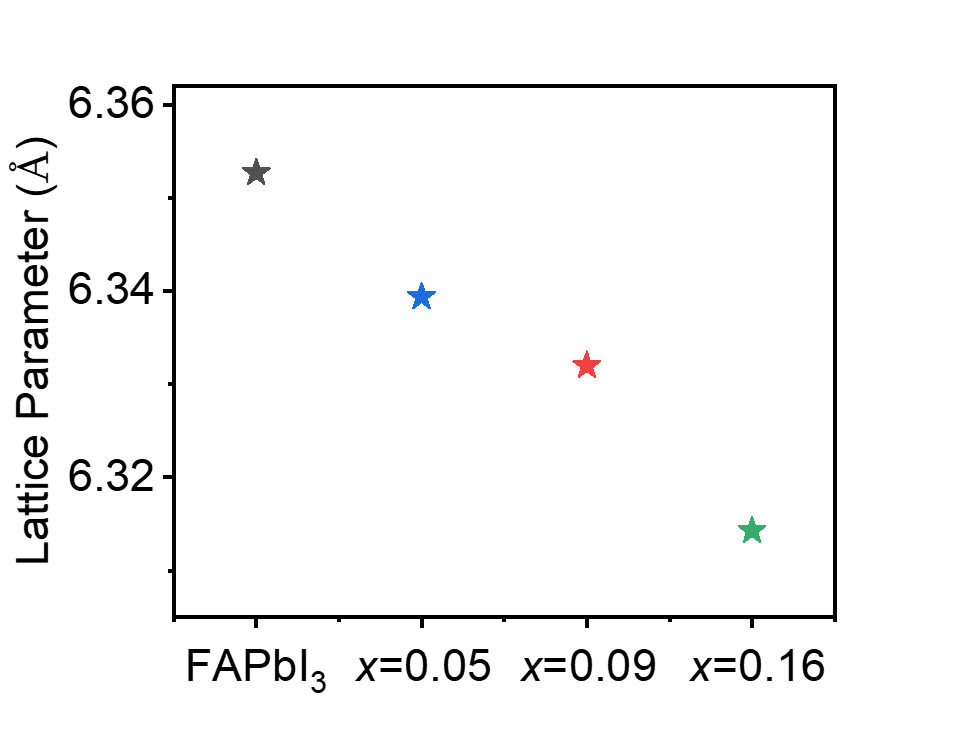


**Supplementary Fig.1 |**The lattice parameter of FAPbI_3_ and SCI-FA_1-_*_x_*Cs*_x_*PbI_3_ (*x*=0.05, 0.09, 0.16) perovskites extracted from (001) perovskite crystal planes in XRD.


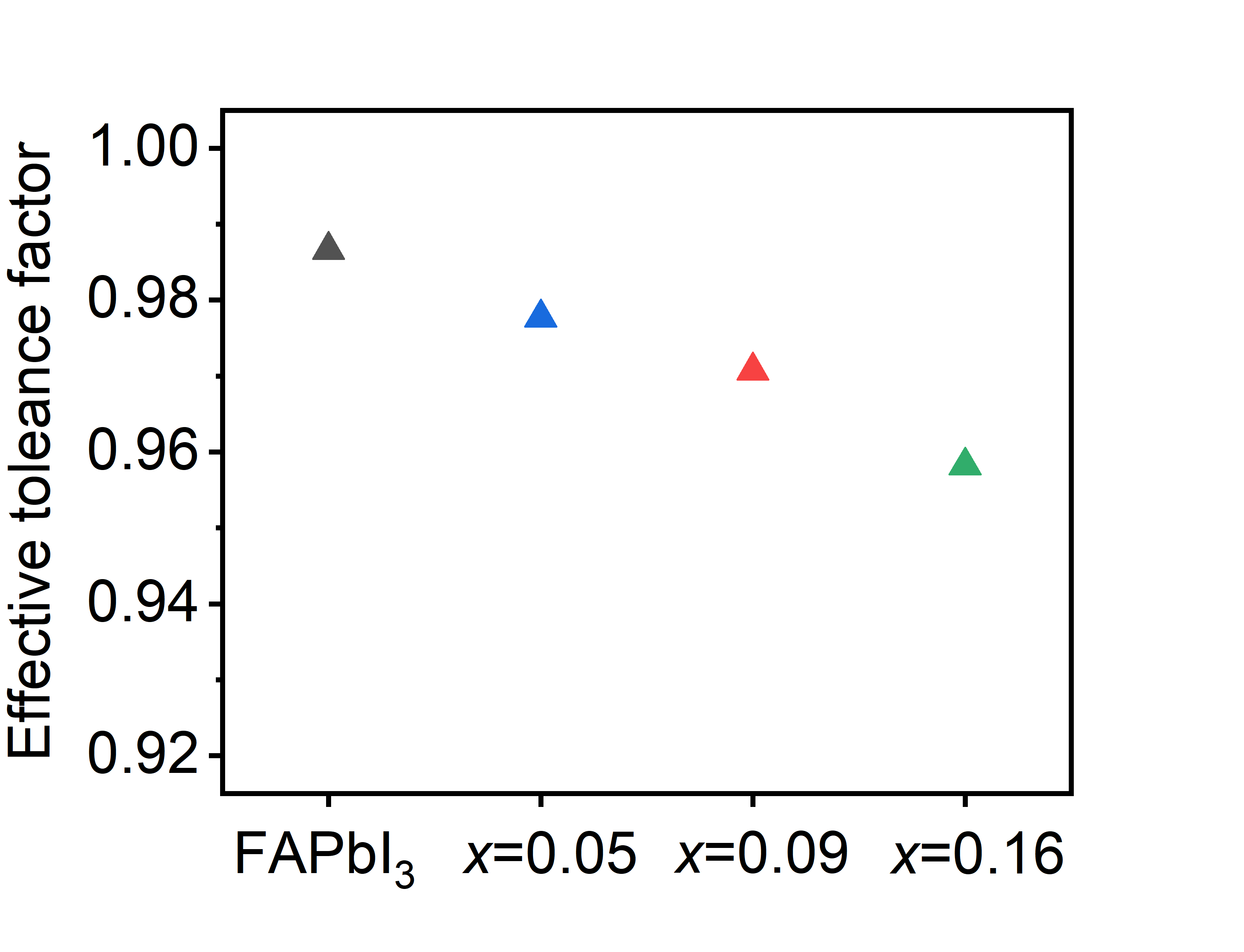


**Supplementary Fig.2 |**The effective tolerance factors of FAPbI_3_ and SCI-FA_1-_*_x_*Cs*_x_*PbI_3_ (*x*=0.05, 0.09, 0.16) perovskites.


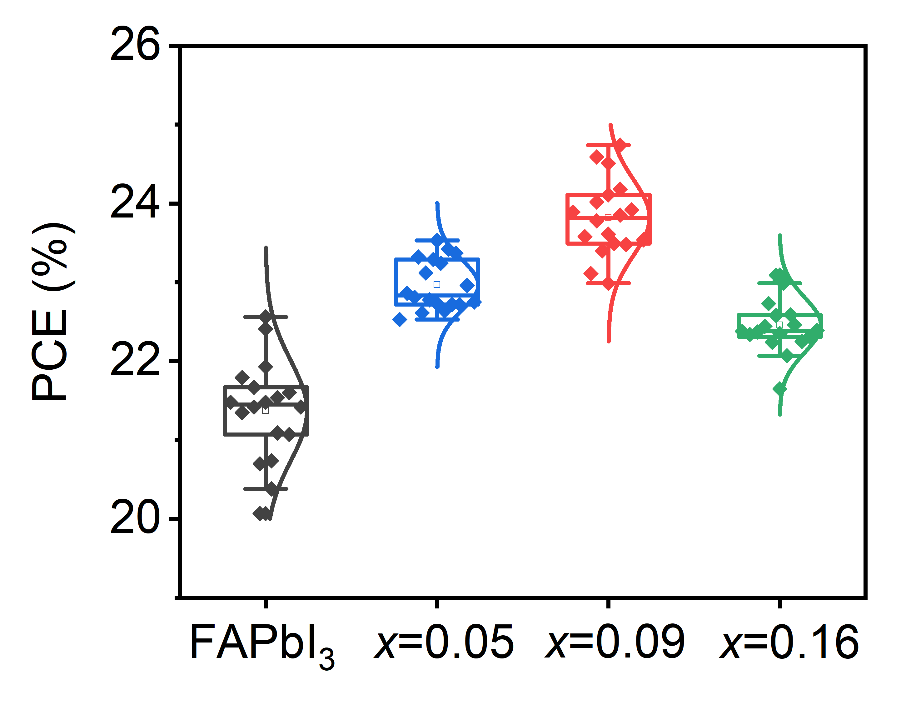


**Supplementary Fig.3 |**PCE statistics of FAPbI_3_ and SCI-FA_1-_*_x_*Cs*_x_*PbI_3_ (*x*=0.05, 0.09, 0.16) PSCs.


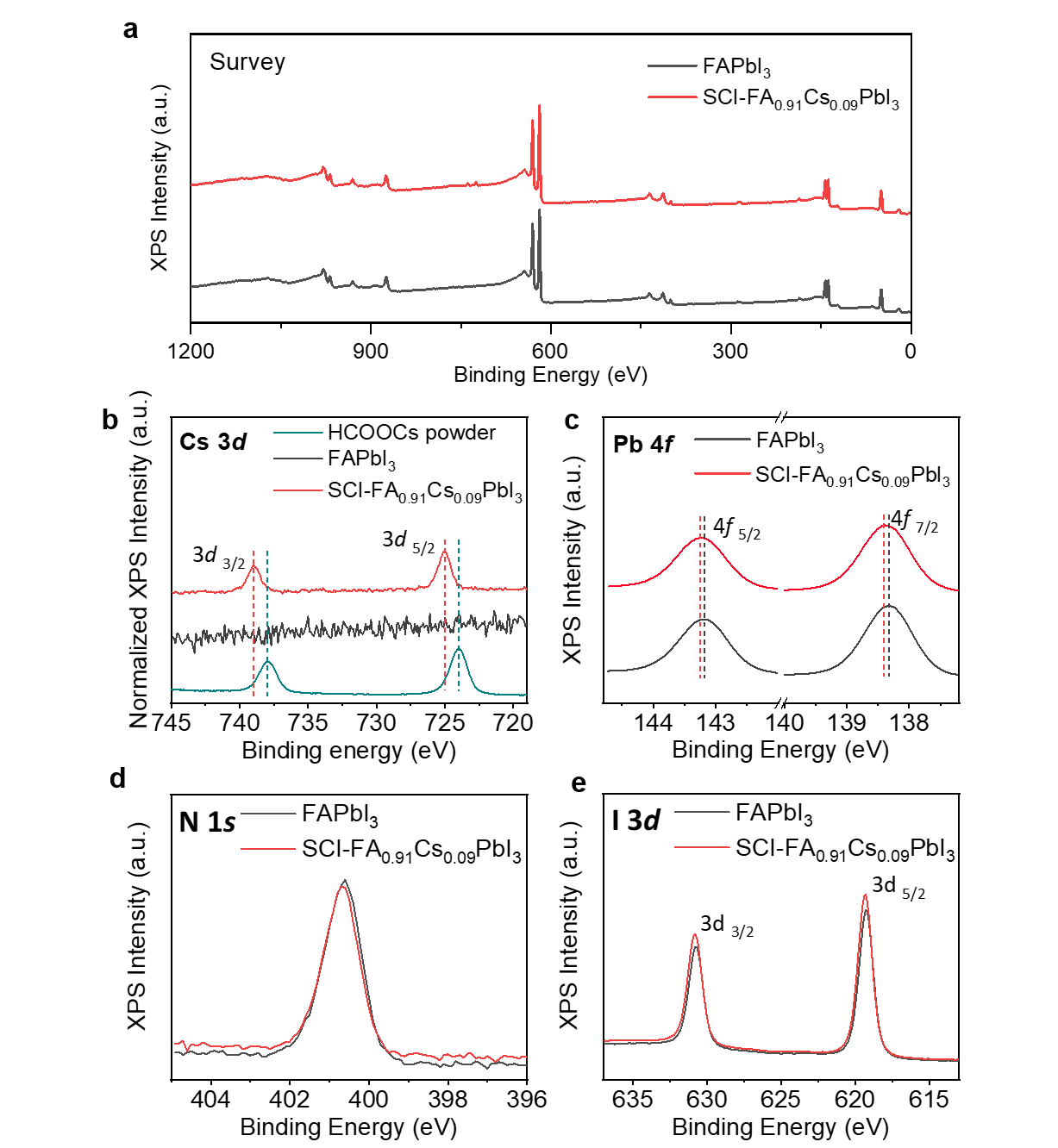


**Supplementary Fig.4 |** **a**. Survey XPS (using Al-K with photon energy of 1486.6 eV) and high resolution Cs 3d (**b**), Pb 4f (**c**), N 1s (**d**) and I 3d (**e**) core-level spectra for FAPbI_3_ and SCI-FA_0.91_Cs_0.09_PbI_3_ perovskites films.


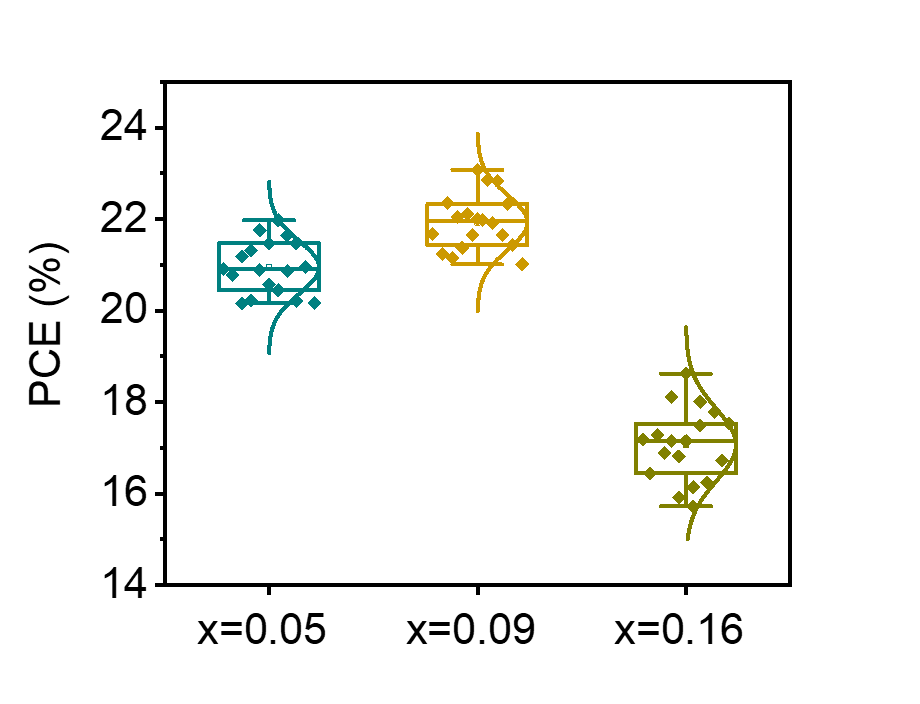


**Supplementary Fig. 5 |**PCE statistics of 1S-FA_1-_*_x_*Cs*_x_*PbI_3_ (*x*=0.05, 0.09, 0.16) PSCs.


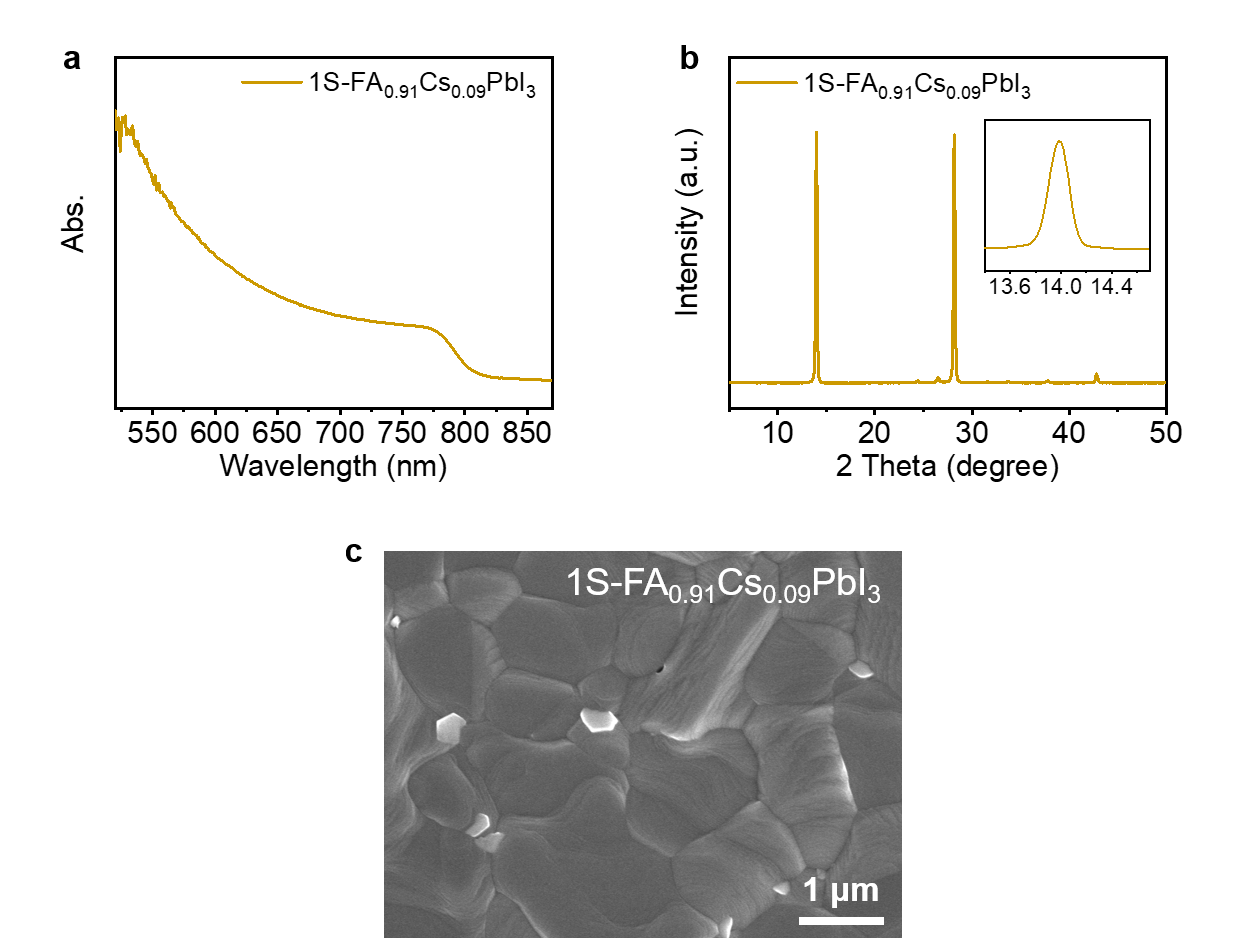


**Supplementary Fig. 6 |** UV-vis absorption, XRD patterns and top-surface images of 1S-FA_0.91_Cs_0.09_PbI_3_ perovskites, inset pattern corresponding to (001) perovskite crystal planes characteristic peaks.

**
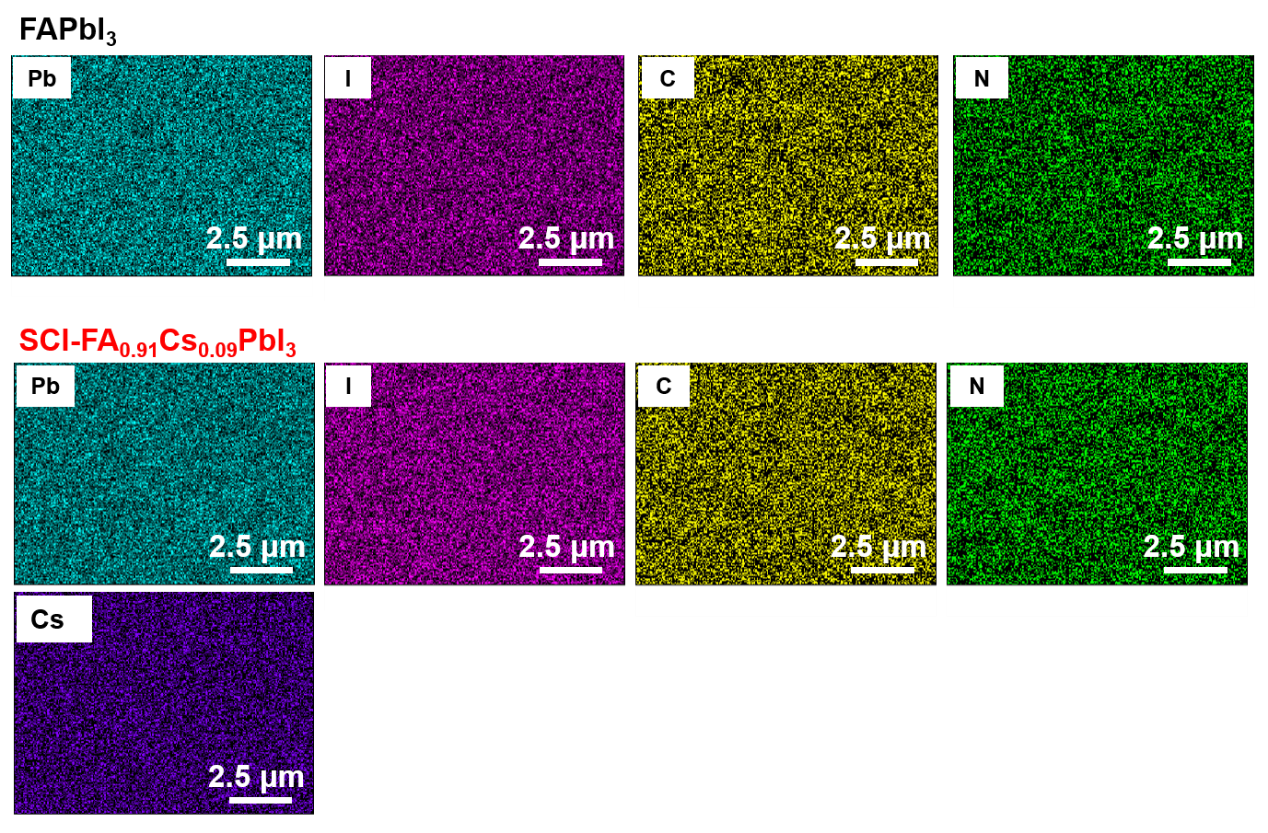
** **Supplementary Fig.7 |**EDS top view element mapping of FAPbI_3_ and SCI-FA_0.91_Cs_0.09_PbI_3_ perovskites films.

**
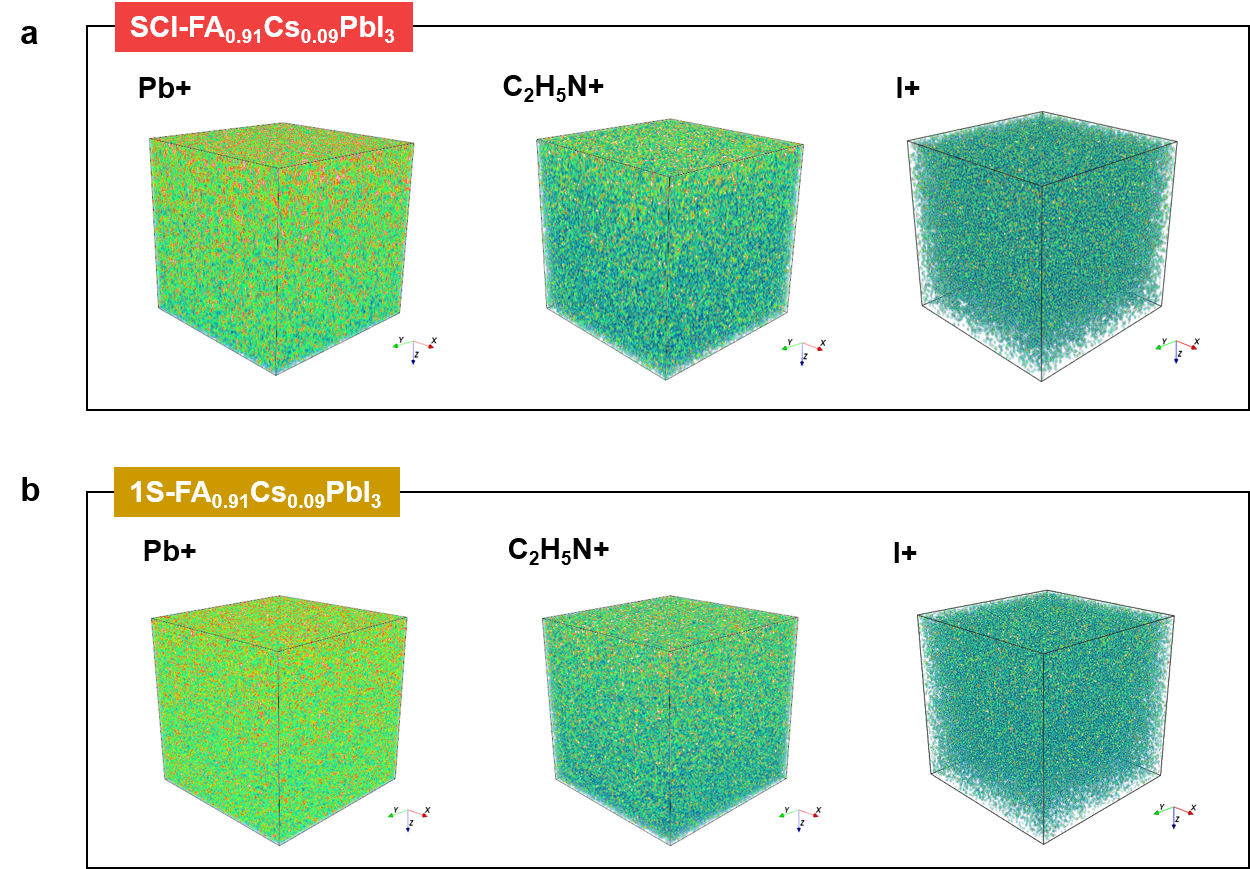
Supplementary Fig.8 |** 3D distribution of Pb^+^, FA^+^, and I^+^ in SCI-FA_0.91_Cs_0.09_PbI_3_ and 1S-FA_0.91_Cs_0.09_PbI_3_ perovskite films under positive polarity by ToF-SIMS.


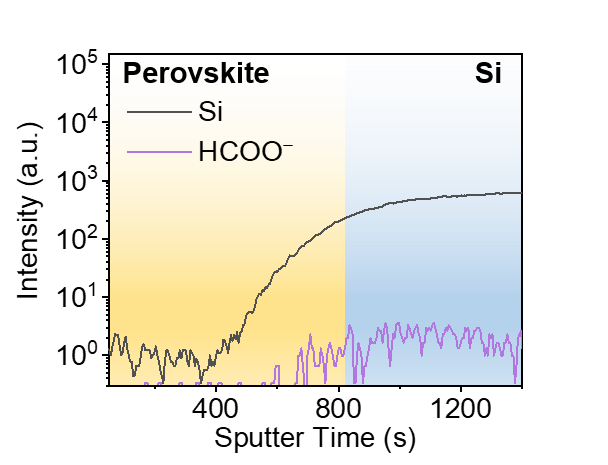


**Supplementary Fig. 9 |** The distribution of HCOO^−^ in the SCI-FA_0.91_Cs_0.09_PbI_3_ film by ToF-SIMS depth profiling analysis.


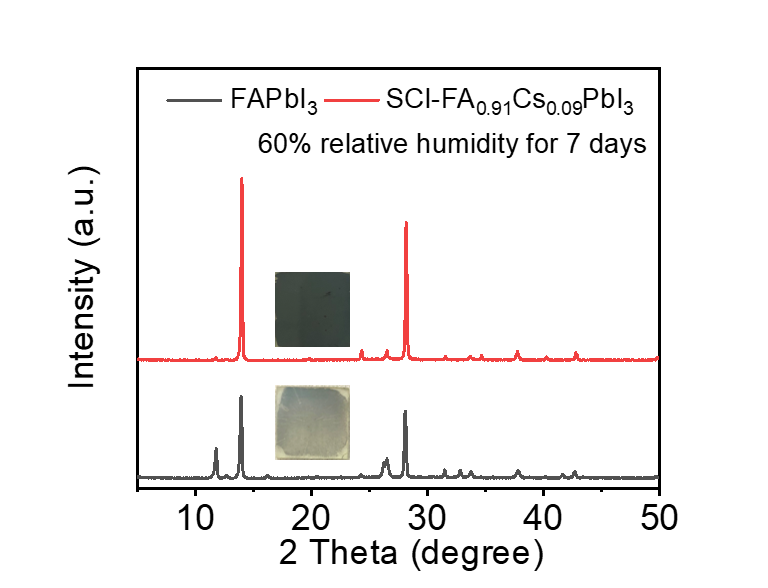


**Supplementary Fig.10 |** Humidity stability of FAPbI_3_ and SCI-FA_0.91_Cs_0.09_PbI_3_ perovskite films, which are exposed in 60% relative humidity for 7 days.


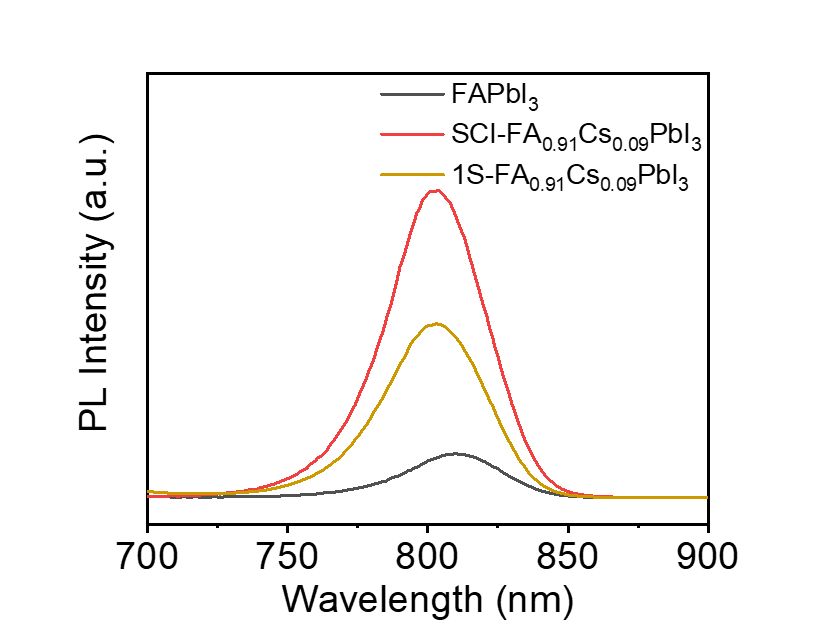


**Supplementary Fig.11 |** PL spectra of FAPbI_3_, SCI-FA_0.91_Cs_0.09_PbI_3_ and 1S-FA_0.91_Cs_0.09_PbI_3_ perovskites films.


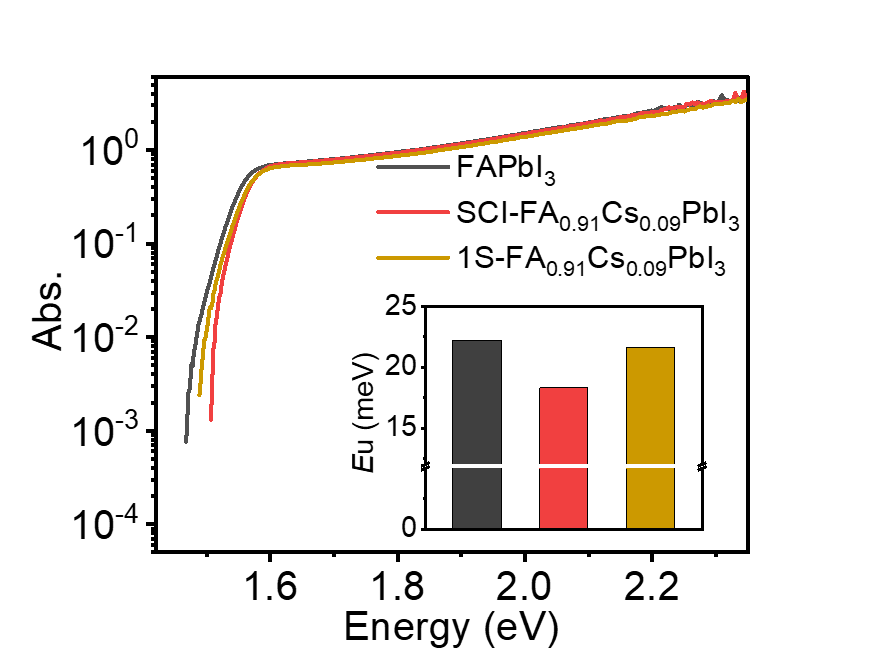


**Supplementary Fig.12 |** Absorption of the FAPbI_3_, SCI-FA_0.91_Cs_0.09_PbI_3_ and 1S-FA_0.91_Cs_0.09_PbI_3_ films. The inset shows the average Urbach energies for these samples.


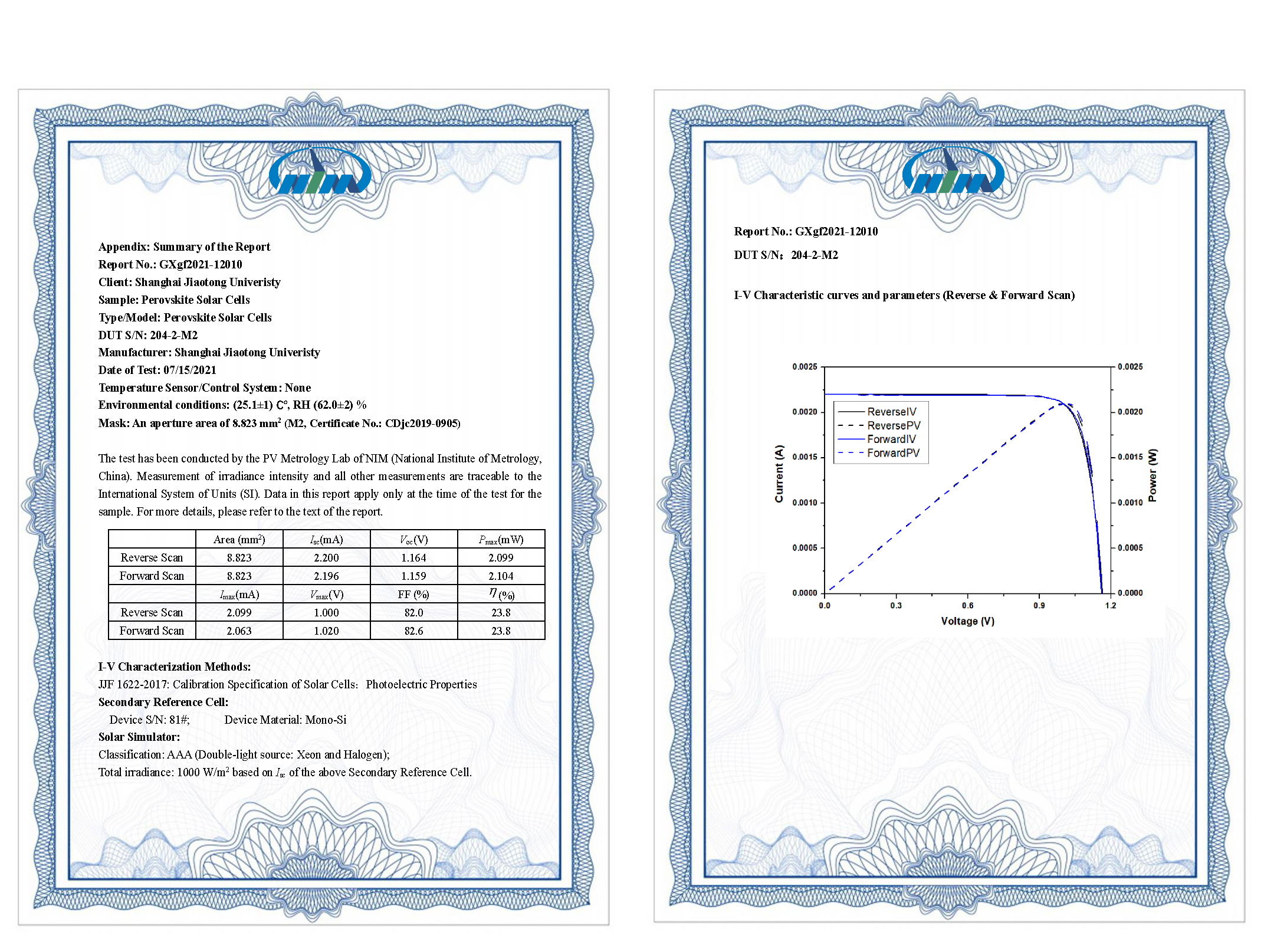
 **Supplementary Fig.13 |**An independent third party PCE certification report of a SCI-FA_0.91_Cs_0.09_PbI_3_-based PSC device by an accredited PV Metrology Laboratory of NIM (National Institute of Metrology, China) verified a PCE of 23.8% (reverse scan, a *I*_sc_ of 2.200 mA, a *V*_oc_ of 1.164 V, and a *FF* of 82.0% and forward scan, a *I*_sc_ of 2.196 mA, a *V*_oc_ of 1.159 V, and a FF of 82.6%). The cell was tested in air without encapsulation or protection during the testing process.

*
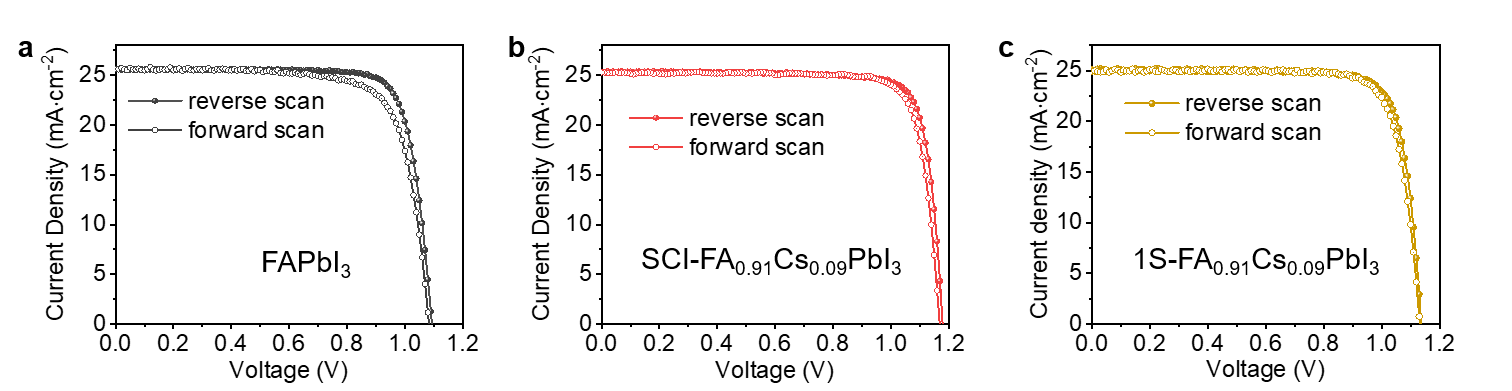
*

**Supplementary Fig.14 |** *J-V* curves under both reverse and forward scan directions of FAPbI_3_, SCI-FA_0.91_Cs_0.09_PbI_3_ and 1S-FA_0.91_Cs_0.09_PbI_3_ based PSCs.


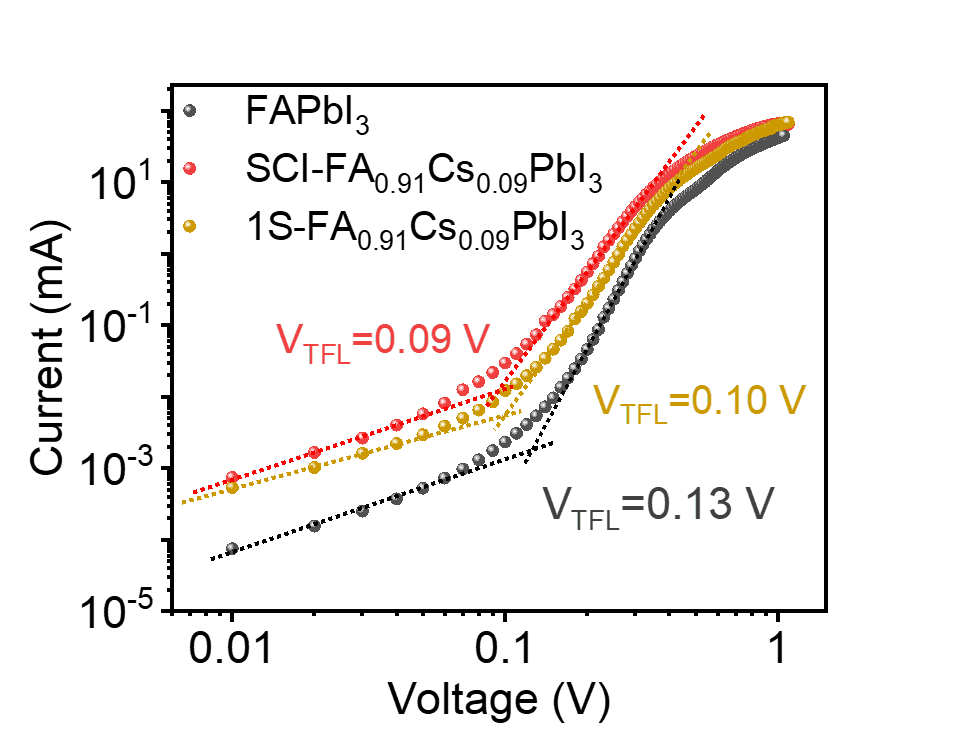


**Supplementary Fig.15 |** Dark I-V curves based on FAPbI_3_, SCI-FA_0.91_Cs_0.09_PbI_3_ and 1S-FA_0.91_Cs_0.09_PbI_3_ electron-only devices.


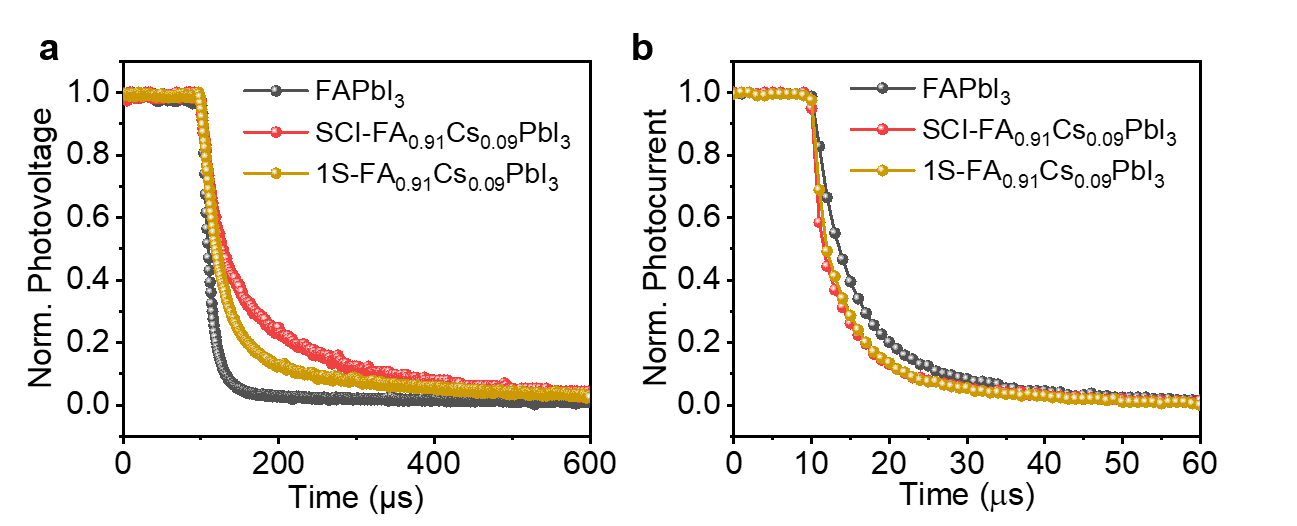


**Supplementary Fig.16 |** **a.** TPV and **b.** TPC of FAPbI_3_, SCI-FA_0.91_Cs_0.09_PbI_3_ and 1S-FA_0.91_Cs_0.09_PbI_3_ based PSCs.

**
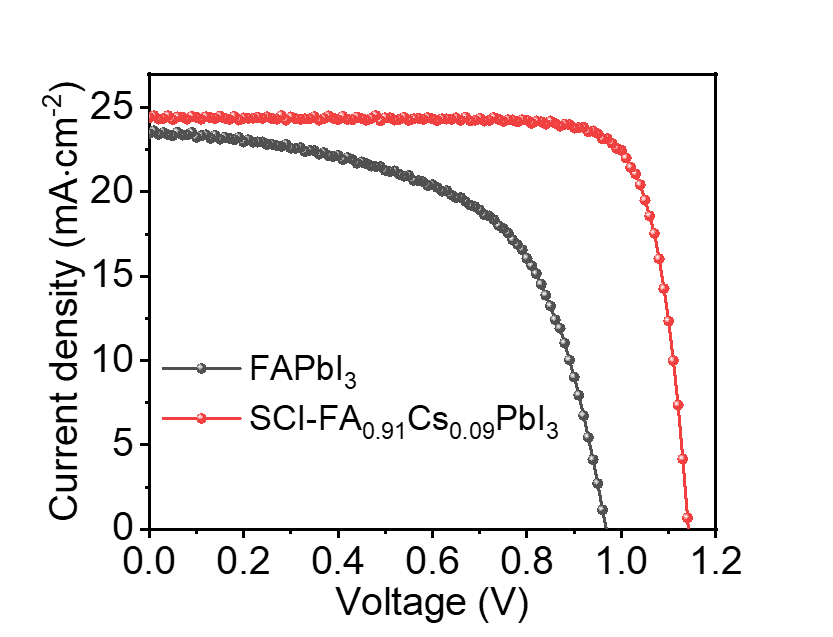
**

**Supplementary Fig.17 |** J-V curves of FAPbI_3_ and SCI-FA_0.91_Cs_0.09_PbI_3_ based PSCs after MPP tracking 1000 h under 1 sun illumination.

**
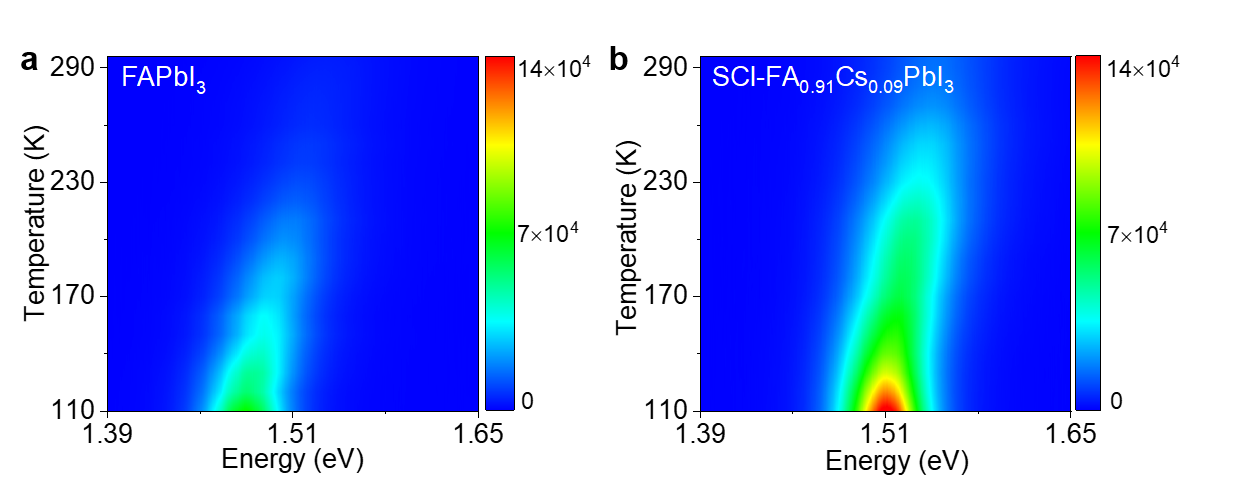
**

**Supplementary Fig.18 |** Temperature-dependent PL spectra of FAPbI_3_ and SCI-FA_0.91_Cs_0.09_PbI_3_ films.

The temperature-dependent PL line width of band-to-band transitions within semiconductors is relatively well understood, being described by the following equation:

$$\Gamma(T)=\Gamma_{\text{inh}}+\frac{\Gamma_{\text{LO}}}{e^{\frac{h\omega}{k_{B}T}}-1}$$

in which the *Γ*_inh_ is the inhomogeneous line width due to structural disorder. *Γ*_LO_ denotes the electron-LO phonon coupling coefficient, and *hω* represents the LO phonon energy.

**Supplementary Table 1** **|** ICP-MS results of soaking SCI-FA_1-_*_x_*Cs*_x_*PbI_3_ perovskite samples. Every perovskite film sample is deposited onto FTO substrate and immersed in 4mL 1M dilute hydrochloric acid. There are three parallel samples for each condition.

| HCOOCs@IPA | Concentration of  Cs (ppm) | Concentration of  Pb (ppm) | n_Cs_: n_Pb_ |
| --- | --- | --- | --- |
| 2.5 mg mL^-1^ | 2.068 | 69.336 | 0.0527±0.55% |
|  | 1.74 | 49.208 |  |
|  | 2.036 | 55.924 |  |
| 5 mg mL^-1^ | 3.436 | 59.234 | 0.0928±0.29% |
|  | 3.568 | 64.579 |  |
|  | 2.548 | 43.112 |  |
| 10 mg mL^-1^ | 5.904 | 56.792 | 0.1606±0.15% |
|  | 6.004 | 59.184 |  |
|  | 5.224 | 66.438 |  |

**Supplementary Table 2 |** The device performance of previously reported pure iodide FA1-xCsx based high efficiency PSCs.

| perovskite | J_SC_  (mA cm^-2^) | V_OC_  (V) | FF | PCE  (%) | ref. |
| --- | --- | --- | --- | --- | --- |
| Rb_5_Cs_10_FAPbI_3_ | 25.06 | 1.08 | 0.755 | 20.44 | Science,2018, 362,449-453 |
| β-GUA-doped  FA_0.95_Cs_0.05_PbI_3_ | 24.41 | 1.14 | 0.796 | 22.2 | Adv. Mater., 2020, 32, 2000571 |
| FA_0.9_Cs_0.1_PbI_3_ | 23.72 | 1.092 | 0.781 | 20.23 | Joule, 2020, 4, 1743-1758 |
| Cs_0.08_FA_0.92_PbI_3_ | 25.86 | 1.11 | 0.81 | 23.25 | Angew. Chem. Int. Ed., 2021, 60, 4238-4244 |
| FA_0.9_Cs_0.1_PbI_3_  (1 cm^2^) | 24.0 | 1.15 | 0.75 | 20.9 | Nat. Commun.,2018, 9, 4482 |
| FA_0.91_Cs_0.09_PbI_3_ | 25.28 | 1.18 | 0.830 | 24.7 | this work |

**Supplementary Table 3 |**Parameters derived via fitting the PL FWHM temperature dependence.

|  | *Γ*_inh_ (meV) | *Γ*_LO_ (meV) | *hω* (meV) |
| --- | --- | --- | --- |
| FAPbI_3_ | 46.1±0.8 | 130.5±15.9 | 35.8±2.6 |
| SCI-FA_0.91_Cs_0.09_PbI_3_ | 32.3±1.6 | 35.3±11.0 | 14.3±3.9 |
